# Isorosthornins A-C, new *ent*-kaurane diterpenoids from *Isodon rosthornii*

**DOI:** 10.1007/s13659-011-0031-7

**Published:** 2011-12-12

**Authors:** Rui Zhan, Xue Du, Jia Su, Xiao-Nian Li, Wei-Guang Wang, Cheng-Qin Liang, Jian-Hong Yang, Yan Li, Jian-Xin Pu, Han-Dong Sun

**Affiliations:** 1 State Key Laboratory of Phytochemistry and Plant Resources in West China, Kunming Institute of Botany, Chinese Academy of Sciences, Kunming, 650201 China; 2 Graduate University of Chinese Academy of Sciences, Beijing, 100049 China

**Keywords:** *Isodon rosthornii*, *ent*-kaurane diterpenoid, cytotoxicity

## Abstract

Three new *ent*-kauranoids, isorosthornins A-C (**1–3**), and a new natural product, dihydroponicidin (**4**), together with five known ones were isolated from the aerial parts of *Isodon rosthornii*. The structures were determined by means of extensive spectroscopic analysis. All diterpenoids isolated were evaluated for their cytotoxicity against HL-60, SMMC-7721, A-549, MCF-7, and SW480 cell lines, and compounds **5** and **7** showed significant inhibitory effects on all cell lines.

**Electronic Supplementary Material:**

Supplementary material is available for this article at 10.1007/s13659-011-0031-7 and is accessible for authorized users.

## Introduction

The genus *Isodon*, which includes about 150 species, is an important genus of the family Lamiaceae. It is widely spread and abundant with diterpenoids which were reported to exhibit antibacterial, anti-inflammatory, and anti-cancer activities.[1] Over the past 30 years, more than 500 diterpenoids were isolated and characterized from over 50 *Isodon* species.[2]

*Isodon rosthornii* (Diels) Hara, a perennial herb, is mainly distributed in Sichuan and Guizhou Provinces of China.[3] It has been used to treat rheumatism and sore throat in folk tradition.[3] Previous phytochemical investigations of this herb led to the discovery of four new *ent*-kauranoids, rosthornins A–D, with antibacterial activity.[4] Due to the secondary metabolites of the genus *Isodon* often differ when grown in different ecological environments, and *I. rosthornii* indigenous to Qionglai City of Sichuan had not been studied on the secondary metabolites before, we explored it with the intent of finding novel and bioactive substances. This investigation led to the isolation of three new *ent*-kaurane diterpenoids, isorothornins A–C, dihydroponicidin (**4**), along with five known ones. This work will describe the isolation, structure determination, and cytotoxic evaluation of these compounds.

## Results and Discussion

The air-dried and powdered aerial parts of *I. rosthornii* was extracted by 70% aqueous acetone, and then partitioned between EtOAc and $H_2O$. The EtOAc solubles were subjected to silica gel, MCI CHP-20 gel, Sephadex LH-20, Lichroprep RP-18 gel column chromatographies (CC), and semipreparative HPLC to afford three new *ent*-kaurane diterpenoids, isorothornins A–C (**1**–**3**), a new natural product, dihydroponicidin (**4**)[5], and five known compounds. The known compounds were identified as ponicidin (**5**)[5], macrocalin B (**6**)[6], xerophilusin B (**7**)[6], rabdonervosin A (**8**)[7], rabdonervosin B (**9**)[8]. The structures of the known compounds were determined by comparing spectroscopic data with literature values.

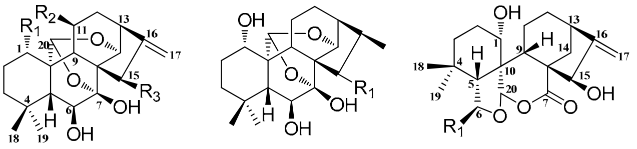

| | R1 | R2 | R3 |
|---|---|---|---|
| **1** | OH | H | OH |
| **5** | OH | H | =O |
| **6** | H | OH | =O |
| **7** | H | H | =O |

| | R1 |
|---|---|
| **2** | *β*-OH |
| **3** | *α*-OH |
| **4** | =O |

| | R1 |
|---|---|
| **8** | OMe |
| **9** | OH |

Compound **1** was isolated as white amorphous powder. The molecular formula was determined as $C_{20}H_{28}O_6$ by HRESIMS (quasi-molecular ion peak at *m/z* 387.1792 $[M + Na]^+$), indicating of seven degrees of unsaturation. The IR spectra of **1** showed absorptions at 3500, 1629 $cm^{-1}$, indicating the existence of OH and C=C groups. The $^{13}C$ NMR and DEPT spectra showed typical signals of a 7*α*,20:14*α*,20-diepoxy-*ent*-kaurane: three methines (C-5, C-9, C-13); three quaternary carbon (C-4, C-8, C-10); characteristic signals of a hemiketal quaternary carbon C-7 ($\delta_C$ 101.3) and two oxymethines C-20 ($\delta_C$ 96.9) and C-14 ($\delta_C$ 70.7) (Table 1). In the $^{13}C$ NMR and DEPT spectra, another three oxymethines and two olefinic (one was a quaternarycarbon, the other was a methlyene) carbons were observed. Detail analyses of $^1H$-$^1H$ COSY

*To whom correspondence should be addressed. E-mail: pujianxin@mail.kib.ac.cn; hdsun@mail.kib.ac.cn

**Table 1.** $^{1}$**H and** $^{13}$**C NMR data of compounds 1 and 2 (500, 125 MHz, in** $C_5D_5N$**,** $\delta$ **ppm,** $J$ **in Hz)***

| | 1 | | 2 | |
|---|---|---|---|---|
| position | $\delta_H$ | $\delta_C$ | $\delta_H$ | $\delta_C$ |
| 1 | 3.89 (m, $H_\beta$) | 72.6 CH | 3.89 (m, $H_\beta$) | 72.8 CH |
| 2 | 1.98 (m) | 30.2 $CH_2$ | 1.97 (m) | 30.1 $CH_2$ |
| 3 | 1.30 (overlapped, $H_\alpha$); 1.27 (m, $H_\beta$) | 40.0 $CH_2$ | 1.40 (m, $H_\alpha$); 1.37 (overlapped, $H_\beta$) | 40.0 $CH_2$ |
| 4 | | 33.4 C | | 33.4 C |
| 5 | 1.62 (br. s, $H_\beta$) | 64.5 CH | 1.58 (br. s, $H_\beta$) | 64.5 CH |
| 6 | 4.16 (d, 4.3) | 72.7 CH | 4.13 (br. s) | 72.9 CH |
| 7 | | 101.3 C | | 101.9 C |
| 8 | | 51.1 C | | 52.6 C |
| 9 | 3.03 (br. s, $H_\beta$) | 44.0 CH | 2.75 (d, 6.0, $H_\beta$) | 40.2 CH |
| 10 | | 48.5 C | | 48.4 C |
| 11 | 2.31 (m) | 19.4 $CH_2$ | 2.30 (overlapped) | 19.3 $CH_2$ |
| 12 | 2.84 (overlapped, $H_\alpha$); 1.56 (m, $H_\beta$) | 28.2 $CH_2$ | 2.49 (overlapped, $H_\alpha$); 1.59 (overlapped, $H_\beta$) | 20.6 $CH_2$ |
| 13 | 2.83 (m, $H_\alpha$) | 40.0 CH | 2.28 (overlapped, $H_\alpha$) | 41.1 CH |
| 14 | 4.75 (d, 6.0, $H_\beta$) | 70.7 CH | 4.68 (d, 5.5, $H_\beta$) | 72.3 CH |
| 15 | 5.42 (s, $H_\alpha$) | 76.5 CH | 5.17 (d, 10.5, $H_\alpha$) | 68.3 CH |
| 16 | | 155.7 C | 2.50 (overlapped) | 36.6 CH |
| 17 | 5.54 (s); 5.28 (s) | 109.5 $CH_2$ | 1.37 (d, 13.5) | 10.0 $CH_3$ |
| 18 | 0.99 (s) | 30.9 $CH_3$ | 0.97 (s) | 30.9 $CH_3$ |
| 19 | 0.91 (s) | 23.2 $CH_3$ | 0.91 (s) | 23.0 $CH_3$ |
| 20 | 5.88 (s) | 96.9 CH | 5.85 (s) | 96.8 CH |
| 1-OH | 5.34 (br. s) | | | |
| 6-OH | 5.56 (s) | | | |
| 15-OH | 8.97 (s) | | | |

*The data of compounds **1** and **2** were assigned based on DEPT, HSQC, HMBC, COSY and ROESY experiments.

(Fig. 1), HSQC, HMBC (Fig. 1) spectra helped furnish the planar structure of compound **1**. HMBC correlations from H-20 ($\delta_H$ 5.88) to C-7 ($\delta_C$ 101.3) and C-14 ($\delta_C$ 70.7) further verified **1** to be a 7$\alpha$,20:14$\alpha$,20-diepoxy-*ent*-kaurane. The HMBC correlations from H-5 ($\delta_H$ 1.62) and H-9 ($\delta_H$ 3.03) to C-1 ($\delta_C$ 72.6), H-5 ($\delta_H$ 1.62) to C-6 ($\delta_C$ 72.7), $H_2$-17 ($\delta_H$ 5.28 and 5.54) to C-15 ($\delta_C$ 76.5) led to the assignment of OH groups at C-1, C-6, C-15, respectively. Therefore, the gross structure of **1** was determined as 1,6,7,15-tetrahydroxy-7$\alpha$,20:14$\alpha$,20-diepoxy-*ent*-kaur-16-ene.

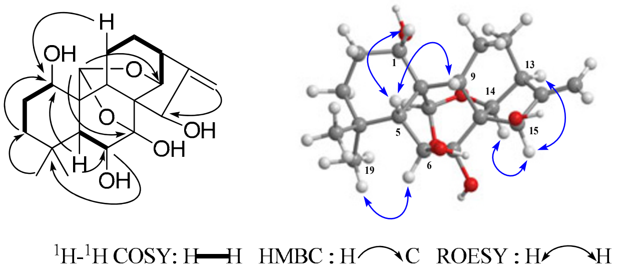

**Fig. 1.** $^1$H-$^1$H COSY, selected HMBC and ROESY correlations of **1**.

In ROESY spectra, H-1 correlated to H-5$\beta$ and H-9$\beta$, H-6 to Me-19, H-15 to H-13$\alpha$ and H-14$\beta$, revealed H-1, H-6, H-15 to be $\beta$-,$\alpha$-,$\alpha$-oriented, respectively (Fig. 1). Thus, compound **1** was determined to be 1$\alpha$,6$\beta$,7$\beta$,15$\beta$-tetrahydroxy-7$\alpha$,20:14$\alpha$,20-diepoxy-*ent*-kaur-16-ene.

Compounds **2** and **3** had the same molecular formula $C_{20}H_{30}O_6$ on the basis of HRESIMS and NMR data which required six degrees of unsaturation. Comparison of NMR data of **2** and **3** with those of the analogue, ponicidin (**5**)[5], showed that **2** and **3** were similar to ponicidin, and the only difference was that the $\alpha$,$\beta$-unsaturated ketone in ponicidin was reduced in both compounds **2** and **3** (Tables 1 and 2). $^1$H-$^1$H COSY, HSQC, HMBC spectra suggested the same planar structure of **2** and **3**.

Carefully analysis of the ROESY correlation showed that the relative configurations of H-1 and H-6 in both **2** and **3** were the same as those of **5** and the relative configuration of H-16 which was determined by correlation of H-16 and H-14$\beta$ in **2** and **3** were both $\alpha$-oriented. In ROESY spectra of compound **2**, the correlations from H-15 to H-14$\beta$ suggested H-15 to be $\alpha$-oriented, while in compound **3**, the correlations from H-15 to H-9$\beta$ and H-11$\beta$ suggested H-15 to be $\beta$-oriented (Fig. 2). Therefore, the structures of **2** and **3** were determined as 1$\alpha$,6$\beta$,7$\beta$,15$\beta$-tetrahydroxy-7$\alpha$,20:14$\alpha$,20-diepoxy-*ent*-kaurene and 1$\alpha$,6$\beta$,7$\beta$,15$\alpha$-tetrahydroxy-7$\alpha$,20:14$\alpha$,20-diepoxy-*ent*-kaurene, respectively.

**Table 2.** $^{1}$**H and** $^{13}$**C NMR data of compounds 3 and 4 (500, 125 MHz, in** $C_5D_5N$**,** $\delta$ **ppm,** $J$ **in Hz)***

| position | 3 $\delta_H$ | 3 $\delta_C$ | 4 $\delta_H$ | 4 $\delta_C$ |
|---|---|---|---|---|
| 1 | 3.85 (m, $H_\beta$) | 73.9 CH | 3.89 (m, $H_\beta$) | 72.0 CH |
| 2 | 1.97 (m, $H_\alpha$) | 30.1 $CH_2$ | 1.96 (m, $H_\alpha$) | 30.2 $CH_2$ |
| 3 | 1.41(overlapped, $H_\alpha$); 1.36 (m, $H_\beta$) | 40.0 $CH_2$ | 1.53 (m, $H_\alpha$); 1.27 (m, $H_\beta$) | 39.9 $CH_2$ |
| 4 | | 33.5 C | | 33.5 C |
| 5 | 1.63 (br. s, $H_\beta$) | 64.1 CH | 1.60 (br. s, $H_\beta$) | 63.5 CH |
| 6 | 4.15 (br. s) | 73.6 CH | 4.16 (br. s) | 73.0 CH |
| 7 | | 104.4 C | | 101.9 C |
| 8 | | 47.1 C | | 57.4 C |
| 9 | 2.51 (d, 7.0, $H_\beta$) | 44.7 CH | 2.74 (d, 5.5, $H_\beta$) | 44.5 CH |
| 10 | | 48.8 C | | 48.3 C |
| 11 | 2.27 (overlapped, $H_\beta$); 1.40 (overlapped, $H_\alpha$) | 19.4 $CH_2$ | 2.34 (m, $H_\beta$); 1.48 (overlapped, $H_\alpha$) | 19.6 $CH_2$ |
| 12 | 2.50 (overlapped, $H_\alpha$); 1.50 (m, $H_\beta$) | 19.4 $CH_2$ | 2.72 (overlapped, $H_\alpha$); 1.46 (overlapped, $H_\beta$) | 19.0 $CH_2$ |
| 13 | 2.27 (overlapped, $H_\alpha$) | 40.0 CH | 2.59 (m, $H_\alpha$) | 37.6 CH |
| 14 | 4.89 (d, 5.6, $H_\beta$) | 72.6 CH | 5.01 (br. s, $H_\beta$) | 70.1 CH |
| 15 | 4.32 (d, 5.1, $H_\beta$) | 82.4 CH | | 214.4 C |
| 16 | 2.49 (m) | 45.8 CH | 2.45 (m) | 47.5 CH |
| 17 | 1.15 (d, 6.7) | 13.4 $CH_3$ | 1.14 (d, 7.1) | 9.3 $CH_3$ |
| 18 | 1.05 (s) | 31.0 $CH_3$ | 1.01 (s) | 31.0 $CH_3$ |
| 19 | 0.97(s) | 23.0 $CH_3$ | 0.96 (s) | 23.0 $CH_3$ |
| 20 | 5.84 (s) | 96.5 CH | 5.89 (s) | 97.5 CH |

*The data of compounds **3** and **4** were assigned based on DEPT, HSQC, HMBC, COSY and ROESY experiments.

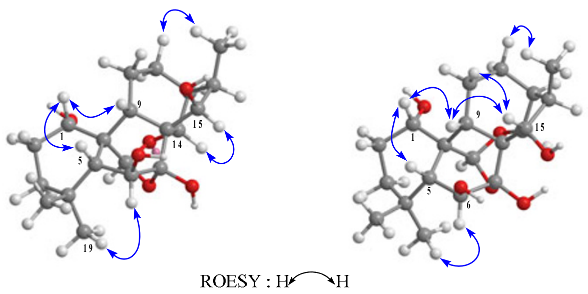

**Fig. 2.** Key ROESY correlations of **2** and **3**.

Compound **4**, white amorphous powder, had the same molecular formula as compound **1** which was verified by HRESIMS (quasi-molecular ion peak at *m/z* 387.1783 [M + Na]$^{+}$). Analysis of its $^{13}$C NMR and DEPT spectrum proved that compound **4** was also a 7$\alpha$,20:14$\alpha$,20-diepoxy-*ent*-kaurane. With the help of HSQC, HMBC, COSY and ROESY experiments, compound **4** was elucidated to be dihydroponicidin[5]. It was a new natural product. Though it was once synthesized, the data of NMR were not reported. $^{1}$H and $^{13}$C NMR data of **4** were listed in Table 2.

Nine diterpenoids isolated were evaluated for cytotoxic activity against five human cancer cell lines (HL-60, A549, SMMC-7721, MCF-7, SW480). The method used was the same as the description in the literature.[9] Compounds **5** and **7** showed significant cytotoxicity against all five cell lines, especially compound **7** ($IC_{50}$ ranged from 1.37 to 3.95 $\mu$M), while compound **6** showed significant cytotoxicity against the cell lines except for A-549 (Table 3). Through analysis of the above bioassay results, a preliminary conclusion that the carbonyl conjugated with an exomethylene group is the active center can be drawn.

**Table 3.** $IC_{50}$ **values (**$\mu$**M) of diterpenoids from** ***I. rosthornii*** **for human tumor cell lines.**

| compound[a] | HL-60 | SMMC-7721 | A-549 | MCF-7 | SW480 |
|---|---|---|---|---|---|
| **5** | 12.5 | 14.8 | 17.1 | 14.4 | 7.3 |
| **6** | 14.7 | 35.5 | > 40 | 28.5 | 9.4 |
| **7** | 1.37 | 2.37 | 3.95 | 2.43 | 2.13 |
| DDP[b] | 1.96 | 16.2 | 17.5 | 17.8 | 12.8 |
| Paclitaxel[b] | < 0.008 | < 0.008 | 1.36 | < 0.008 | 0.04 |

[a]Other selected ones not listed in the table were inactive ($IC_{50}$ > 40 $\mu$M) for all cell lines.
[b]DDP (cisplatin) and paclitaxel were used as positive controls.

## Experimental Section

**General Experimental Procedures**. Petroleum ether (PE, 60–90°C), EtOAc, $CHCl_3$, Acetone, MeOH, EtOH were analytical grade and produced by Sinopharm Chemical Reagent Co. Ltd, China. Column chromatography (CC) was performed on silica gel (200-300 mesh; Qingdao Marine Chemical Inc., Qingdao, China), Lichroprep RP-18 gel (40–63 $\mu$m, Merck, Darmstadt, Germany), and Sephadex LH-20 (Pharmacia). Fractions were monitored by TLC, and spots were visualized by spraying with 10% $H_2SO_4$ in EtOH, followed by heating. Semi-preparative HPLC was performed on an Agilent 1100 HPLC with a Zorbax SB-$C_{18}$, 9.4 mm × 25 cm column. UV data were obtained using a UV-210A spectrometer. IR spectra were obtained on a Bio-Rad FtS-135 spectrophotometer with KBr pellets. HRESIMS were performed on an API QSTAR time-of flight spectrometer. NMR spectra were obtained on

Bruker DRX-500 and DRX-600 instruments with TMS as an internal standard.

**Plant Material.** The aerial parts of *I. rosthornii* were collected in Sichuan province, China, in July 2008, and was identified by Prof. Xi-Wen Li, Kunming Institute of Botany. A voucher specimen (KIB 200809003) has been deposited in the Herbarium of the Kunming Institute of Botany, Chinese Academy of Sciences.

**Extraction and Isolation.** The aerial parts (10 kg) of *I. rosthornii* were extracted with the 70% aqueous acetone (20 L) four times (two days for each time) at room temperature and filtered. The filtrate was evaporated in vacuo to be concentrated. Then the concentrate without acetone (7 L) was portitioned between EtOAc and $H_2O$. The EtOAc soluble portion (485 g) was decolorized on MCI gel and then subjected to silica gel CC (2000 g, 100–200 mesh). Five fractions were given by the silica gel column eluted with a $CHCl_3$/MeOH gradient system (100:1, 50:1, 20:1, 10:1, 5:1, for fraction 1 to 5, respectively). Fraction 1 was chromatographed on normal silica gel column repeatedly (eluted with petroleum ether/acetone 30:1 to 10:1 then $CHCl_3$/MeOH 80:1) and semipreparative HPLC (eluted with 50% MeOH-$H_2O$) to afford dihydroponicidin (**4**, 5.6 mg), xerophilusin B (**7**, 22 mg), rabdonervosin B (**9**, 18 mg). Isolated by normal silica gel column (eluted with $CHCl_3$/MeOH 80:1 to 50:1), RP-18 (30% to 60% MeOH-$H_2O$ ) and semipreparative HPLC (33% acetonitrile-$H_2O$), isorothornin A (**1**, 6.1 mg), rabdonervosin A (**8**, 20 mg) was got from fraction 2, along with isorothornin B (**2**, 6 mg), isorothornin C (**3**, 10 mg), ponicidin (**5**, 1.5 g) and macrocalin B (**6**, 60 mg) from fraction 3.

**Isorothornin A (1):** white amorphous powder; $[\alpha]_D^{26}$ −61.0 (*c* 0.06, MeOH); UV (MeOH) $\lambda_{max}$ (log $\varepsilon$) 201 (0.08) nm; IR (KBr) $\upsilon_{max}$: 3500, 1629, 1089 $cm^{-1}$; $^1H$ and $^{13}C$ NMR data listed in Table 1; HRESIMS (positive): *m/z* 387.1792 ($[M + Na]^+$, $C_{20}H_{28}O_6$, calcd. 387.1783).

**Isorothornin B (2):** white amorphous powder; $[\alpha]_D^{26}$ −118.0 (*c* 0.07, MeOH); UV (MeOH) $\lambda_{max}$ (log $\varepsilon$) 202 (0.21) nm; IR (KBr) $\upsilon_{max}$: 3539, 2944 $cm^{-1}$; $^1H$ and $^{13}C$ NMR data listed in Table 1; HRESIMS (positive): *m/z* 389.1932 ($[M + Na]^+$, $C_{20}H_{30}O_6$, calcd. 389.1940).

**Isorothornin C (3):** white amorphous powder; $[\alpha]_D^{26}$ −81.7 (*c* 0.08, MeOH); UV (MeOH) $\lambda_{max}$ (log $\varepsilon$) 201 (0.04) nm; IR (KBr) $\upsilon_{max}$: 3420, 2928 $cm^{-1}$; $^1H$ and $^{13}C$ NMR data listed in Table 2; HRESIMS (positive): *m/z* 389.1941 ($[M + Na]^+$, $C_{20}H_{30}O_6$, calcd. 389.1940).

**Cytotoxicity Assay.** The following human tumor cell lines were used: HL-60 (human myeloid leukemia cell line), SMMC-7721 (human hepatocarcinoma cell line), A-549 (lung cancer cell line), MCF-7 (breast cancer cell line) and SW480 (human colon carcinoma). All the cells were cultured in RPMI-1640 or DMEM medium (Hyclone, Logan, UT), supplemented with 10% fetal bovine serum (Hyclone, USA) at 37°C in a humidified atmosphere with 5% $CO_2$. Cell viability was assessed by conducting colorimetric measurements of the amount of insoluble formazan formed in living cells based on the reduction of 3-(4,5-dimethylthiazol-2-yl)-2,5-diphenyltetrazolium bromide (MTT) (Sigma, St. Louis, MO). Briefly, 100 $\mu$L adherent cells were seeded into each well of a 96-well cell culture plate and allowed to adhere for 12 h before drug addition, while suspended cells were seeded just before drug addition, both with initial density of $1 \times 10^5$ cells/ml in 100 $\mu$L medium. Each tumor cell line was exposed to the tested compound at various concentrations in triplicates for 48 h, with *cis*platin (Sigma, USA) as positive control. After the incubation, MTT (100 $\mu$g) was added to each well, and the incubation continued for 4 h at 37°C. The cells were lysed with 100 $\mu$L 20% SDS-50% DMF after removal of 100 $\mu$L medium. The optical density of the lysate was measured at 595 nm in a 96-well microtiter plate reader (Bio-Rad 680, USA). The $IC_{50}$ value of each compound was calculated by the Reed and Muench's method.[9]

## Electronic Supplementary Material

Supplementary material is available in the online version of this article at http://dx.doi.org/10.1007/s13659-011-0031-7 and is accessible for authorized users.

## Acknowledgments

This work was supported financially by the NSFC-Joint Foundation of Yunnan Province (No. U0832602 to H. D. Sun), the Major State Basic Research Development Program of China (No. 2009CB522300 and 2009CB940900), the Science and Technology Program of Yunnan Province (No. 2008IF010 and 2008CD162), the NSFC (No. 81172939 to J. X. Pu), and the Major Direction Projection Foundation of CAS Intellectual Innovation Project (No. 2010KIBA05 to J. X. Pu).

**Open Access** This article is distributed under the terms of the Creative Commons Attribution License which permits any use, distribution, and reproduction in any medium, provided the original author(s) and source are credited.

## Electronic supplementary material

Supplementary material, approximately 590 KB.

## References

[1] Sun H. D., Xu Y. L., Jiang B. (2001). Diterpenoids from *Isodon* Species.

[2] Sun H. D., Huang S. X., Han Q. B. (2006). Nat. Prod. Rep..

[3] Academia Sinca. (1979). Botany of China.

[CR4] Hu, K.; Dong, A. J.; Kobayashi, H.; Iwasaki, S.; Yao, X. S. *Plant Deriv. Antimycotics* **2003**, 525–549

[CR5] Kubo I., Xu Y. L., Shimizu K. (2004). Phytother. Res..

[CR6] Li G. Y., Wang Y. L. (1984). Yaoxue Xuebao.

[CR7] Xu Y. L., Li Z. Q. (1998). Yunnan Zhiwu Yanjiu.

[CR8] Xu Y. L., Ma Y. B. (1989). Phytochemistry.

[CR9] Wang X. R., Wang H. P., Hu H. P., Sun H. D., Wang S. Q., Ueda S., Kuroda Y., Fujita T. (1995). Phytochemistry.

[CR10] Fujita, E.; Taoka, M.; Shibuya, M.; Fujita, T.; Shingu, T., *J. Chem. Soc., Perkin Trans. 1* **1973**, 2277–2281.

[6] Hou A. J., Li M. L., Jiang B., Lin Z. W., Ji S. Y., Zhou Y. P., Sun H. D. (2000). J. Nat. Prod..

[7] Gao Y. H., Wu S. H., Zhong R. J., Li G. Y. (1996). Zhongcaoyao.

[8] Gao Y. H., Wu S. H., Cheng Y. (1999). Zhongcaoyao.

[9] Monks A., Scudiero D., Skehan P., Shoemaker R., Paull K., Vistica D., Hose C., Langley P. (1991). J. Natl. Cancer Inst..

