# Supplementary material for: Isorosthornins A-C, new ent-kaurane diterpenoids from Isodon rosthornii
Source: Nat Prod Bioprospect. 2011 Dec 12;1(3):116–20. doi: 10.1007/s13659-011-0031-7 (PMC4131646; doi:10.1007/s13659-011-0031-7)
Supplement: Supplementary file 1 — Supplementary material, approximately 590 KB. [file 13659_2011_31_MOESM1_ESM.pdf]

Electronic Supplementary Material

Isorosthornins A–C, new *ent*-kaurane diterpenoids from *Isodon rosthornii*

Rui ZHAN,[a,b] Xue DU,[a] Jia SU,[a] Xiao-Nian LI,[a] Wei-Guang WANG,[a] Cheng-Qin LIANG,[a] Jian-Hong YANG,[a] Yan LI,[a] Jian-Xin PU,[a,*] and Han-Dong SUN[a,*]

[a]State Key Laboratory of Phytochemistry and Plant Resources in West China, Kunming Institute of Botany, Chinese Academy of Sciences, Kunming 650201, China

[b]Graduate University of Chinese Academy of Sciences, Beijing 100049, China

Received 17 November 2011; Accepted 7 December 2011

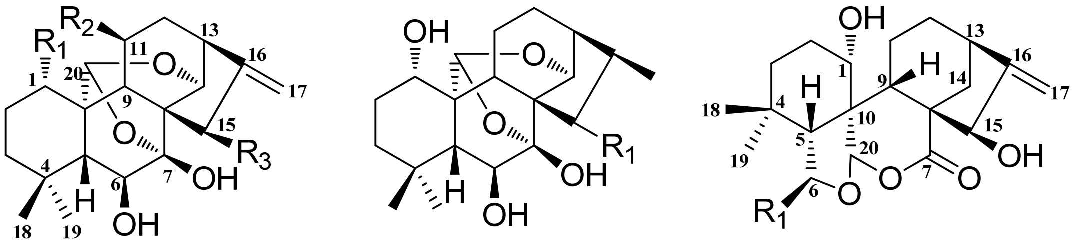

	R_1	R_2	R_3
**1**	OH	H	OH
**5**	OH	H	=O
**6**	H	OH	=O
**7**	H	H	=O

	R_1
**2**	β-OH
**3**	α-OH
**4**	=O

	R_1
**8**	OMe
**9**	OH

Structures of compounds **1**–**9**.

*To whom correspondence should be addressed. E-mail: pujianxin@mail.kib.ac.cn; hdsun@mail.kib.ac.cn

Characterization Data of New Compounds

► **Compound 1：^{1}H, ^{13}C NMR and HRESIMS**

► **Compound 2：^{1}H, ^{13}C NMR and HRESIMS**

► **Compound 3：^{1}H, ^{13}C NMR and HRESIMS**

► **Compound 4：^{1}H, ^{13}C NMR and HRESIMS**

For compound 1:

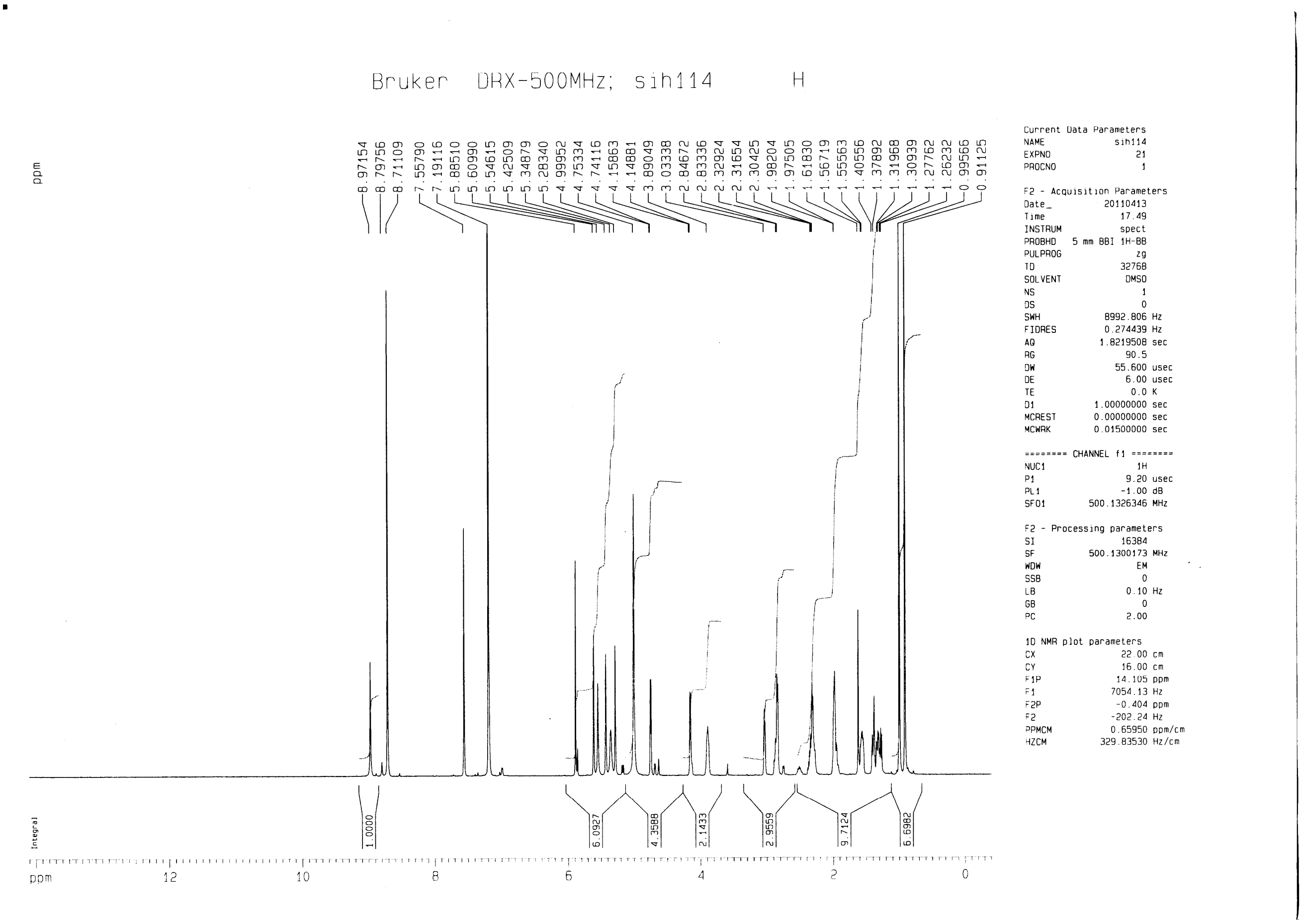

Spectra1 ^{1}H NMR spectrum of compound **1**

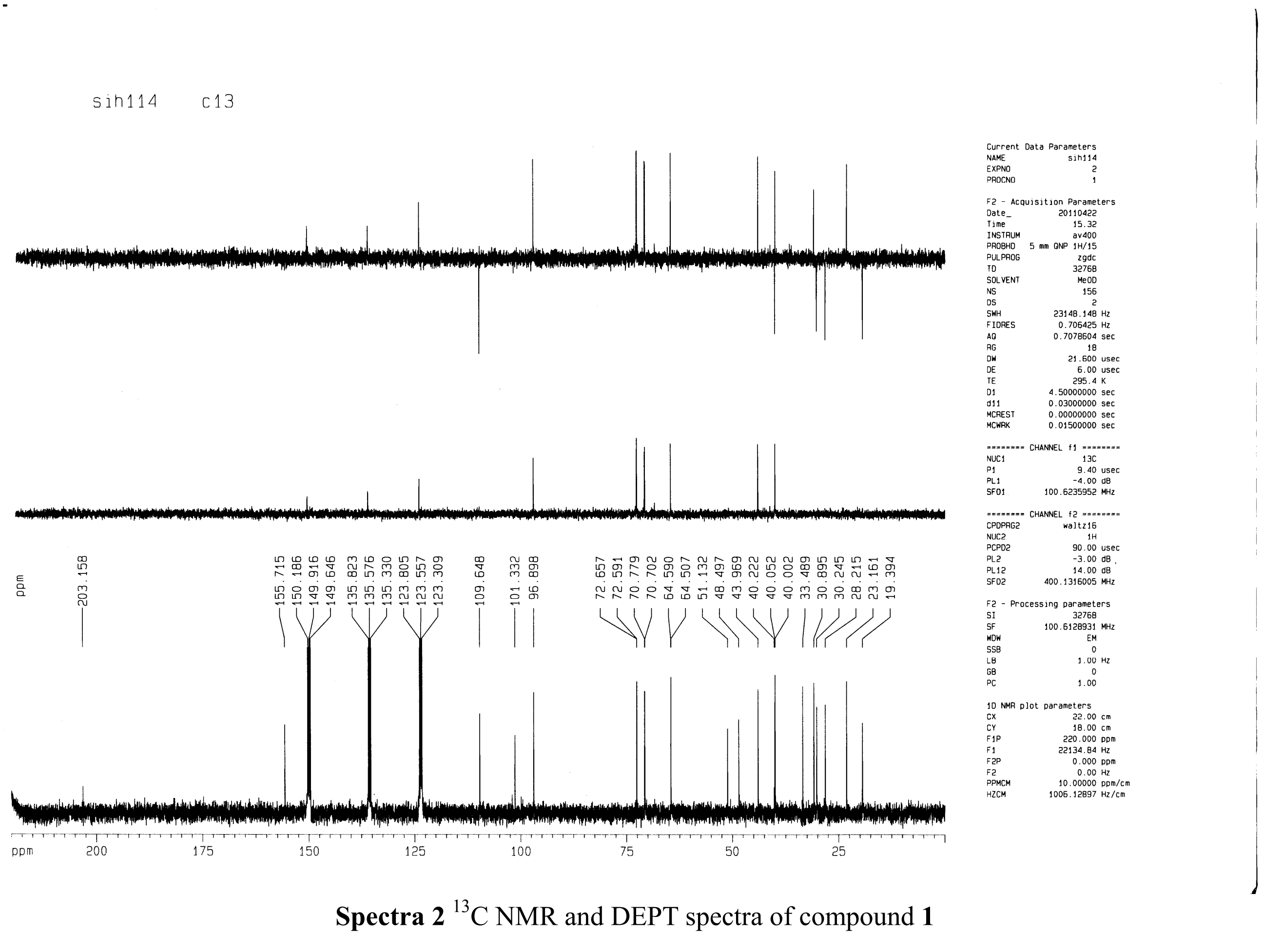

Spectra 2 ^{13}C NMR and DEPT spectra of compound **1**

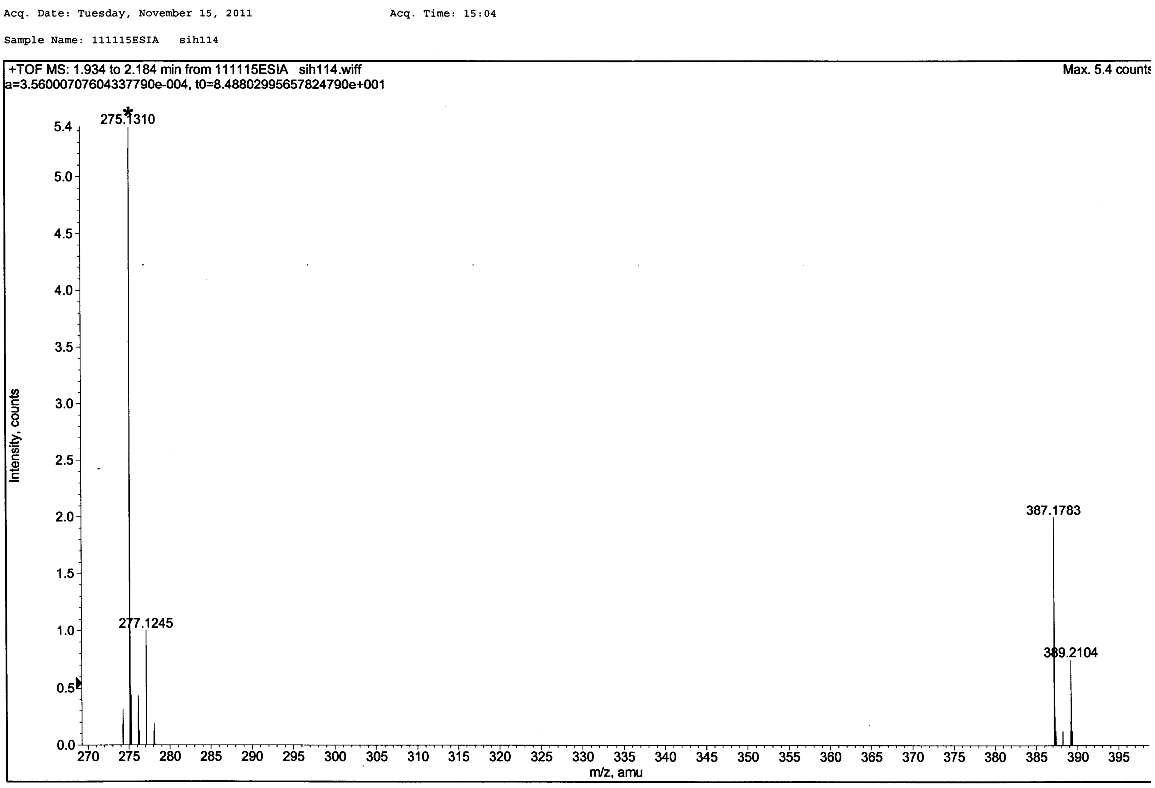

Spectra 3 HRESIMS data of compound **1**

For compound 2

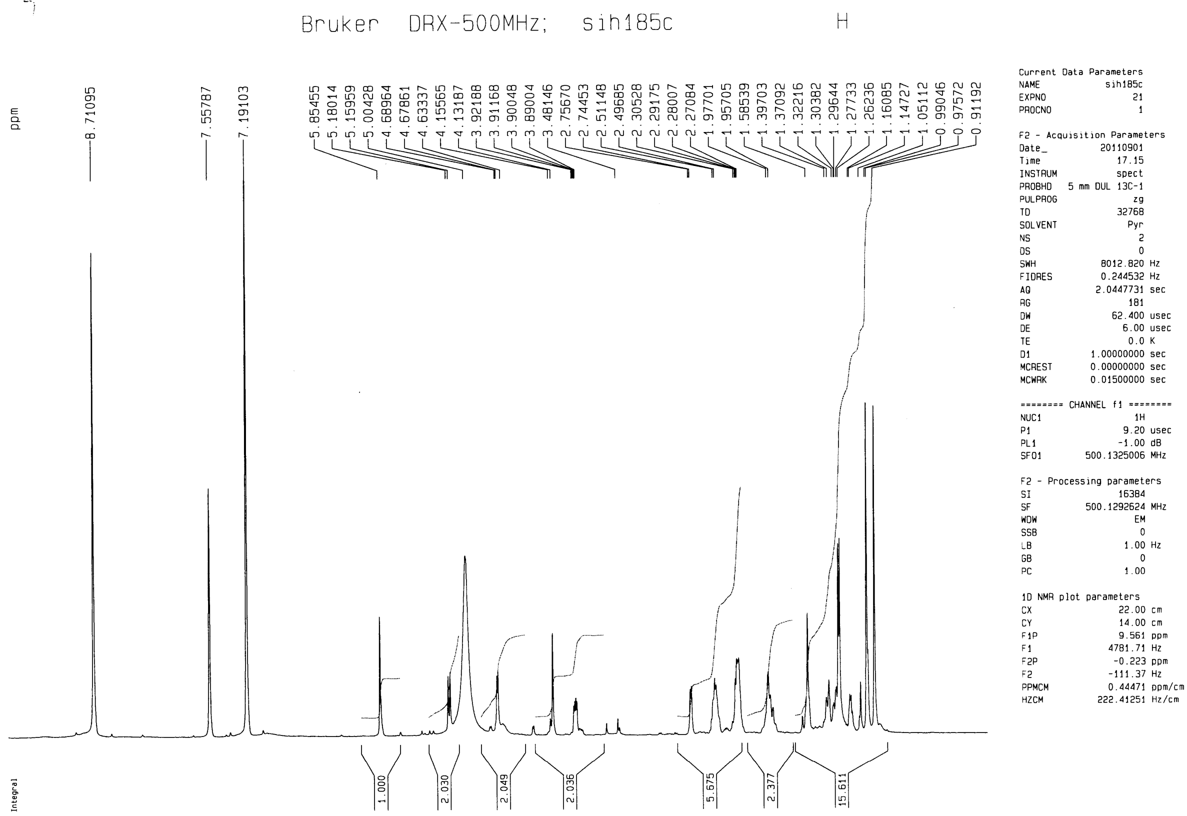

Spectra 4 ^{1}H NMR spectrum of compound **2**.

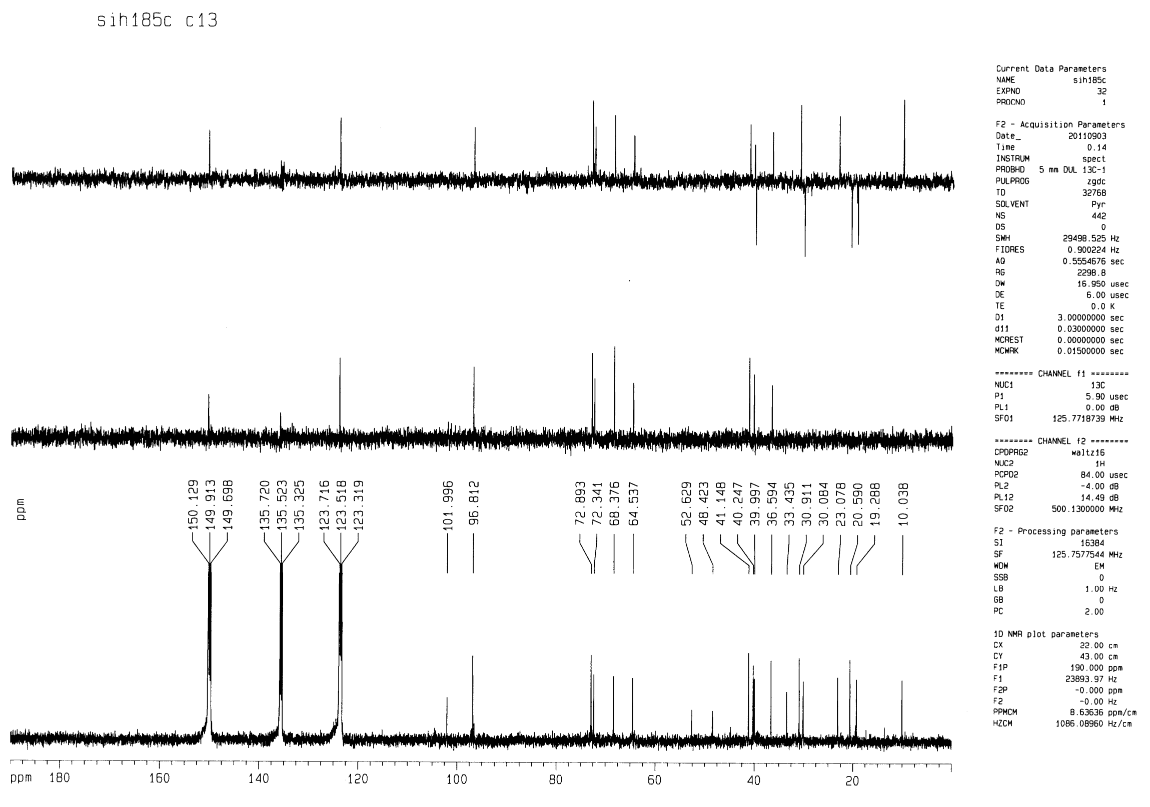

Spectra 5 ^{13}C NMR and DEPT spectra of compound **2**

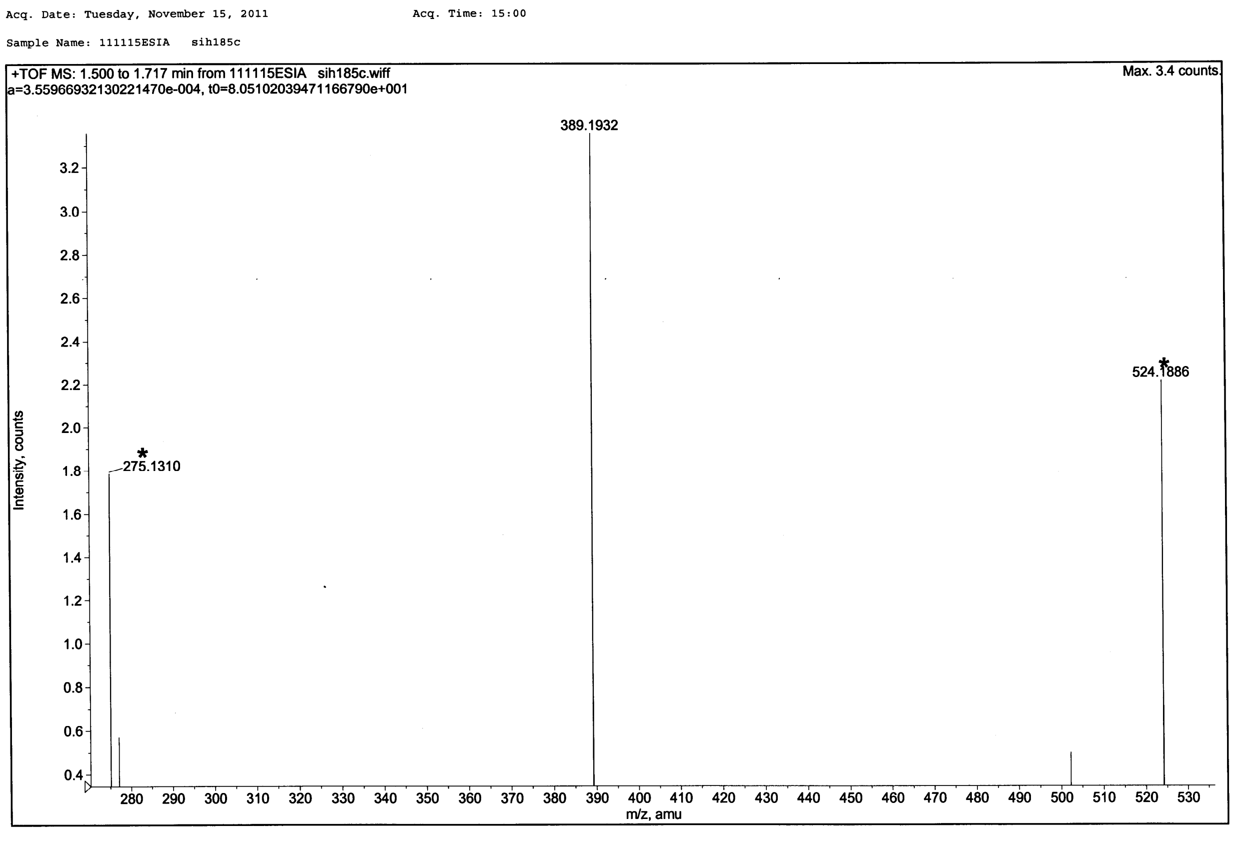

Spectra 6 HRESIMS data of compound **2**

For compound 3

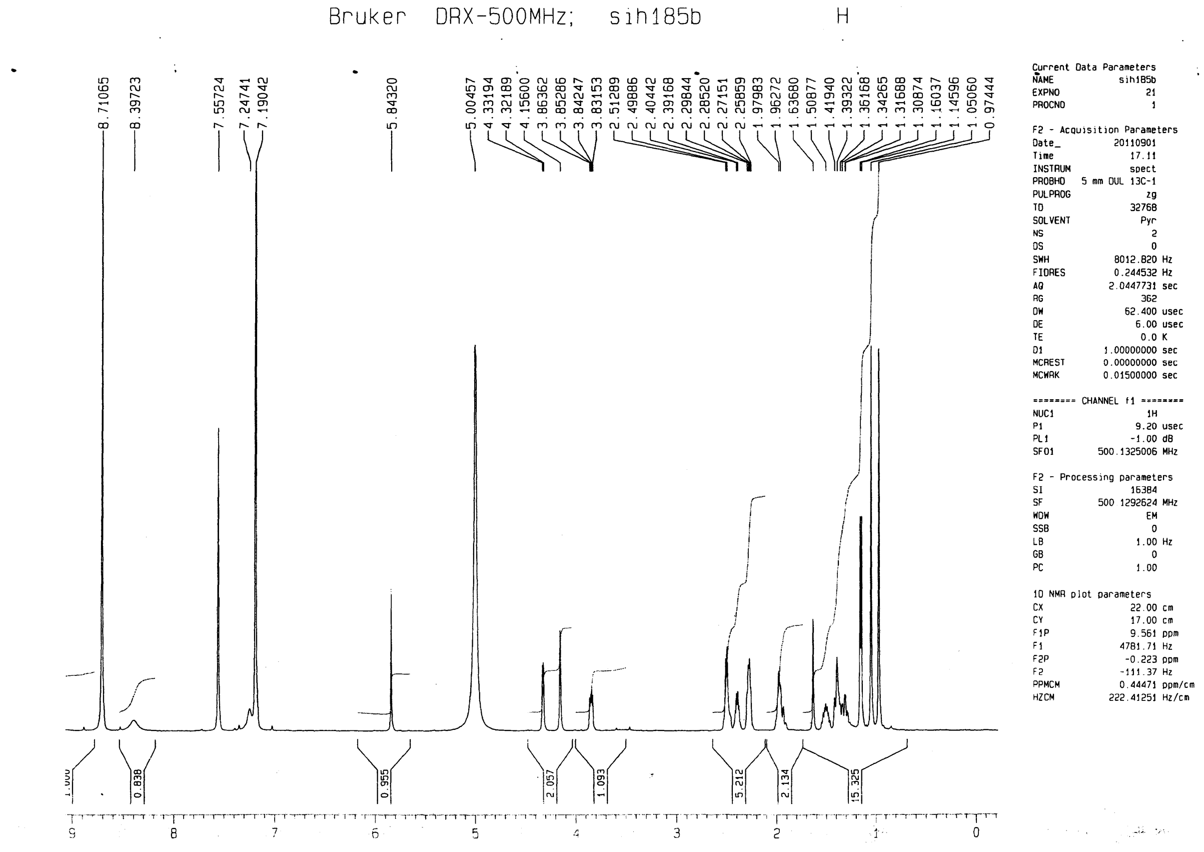

Spectra 7 ^{1}H NMR spectrum of compound **3**

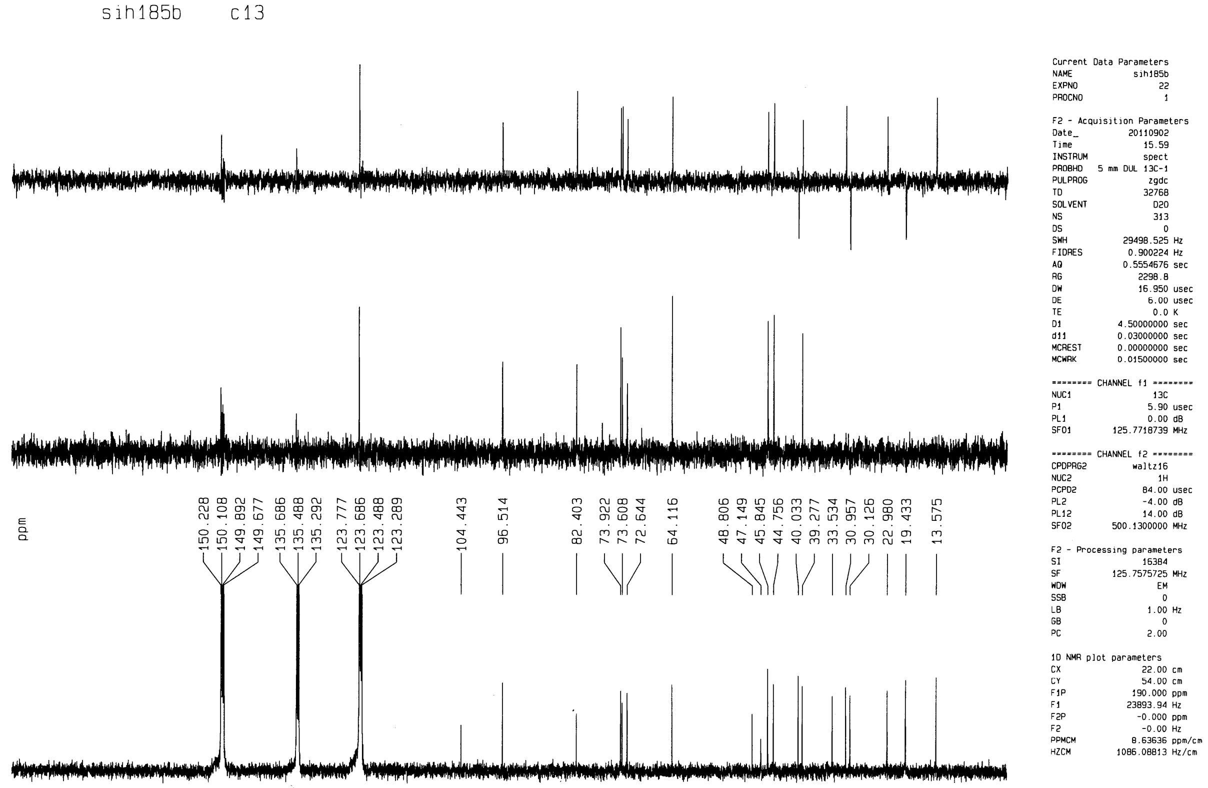

Spectra 8 ^{13}C NMR and DEPT spectra of compound **3**

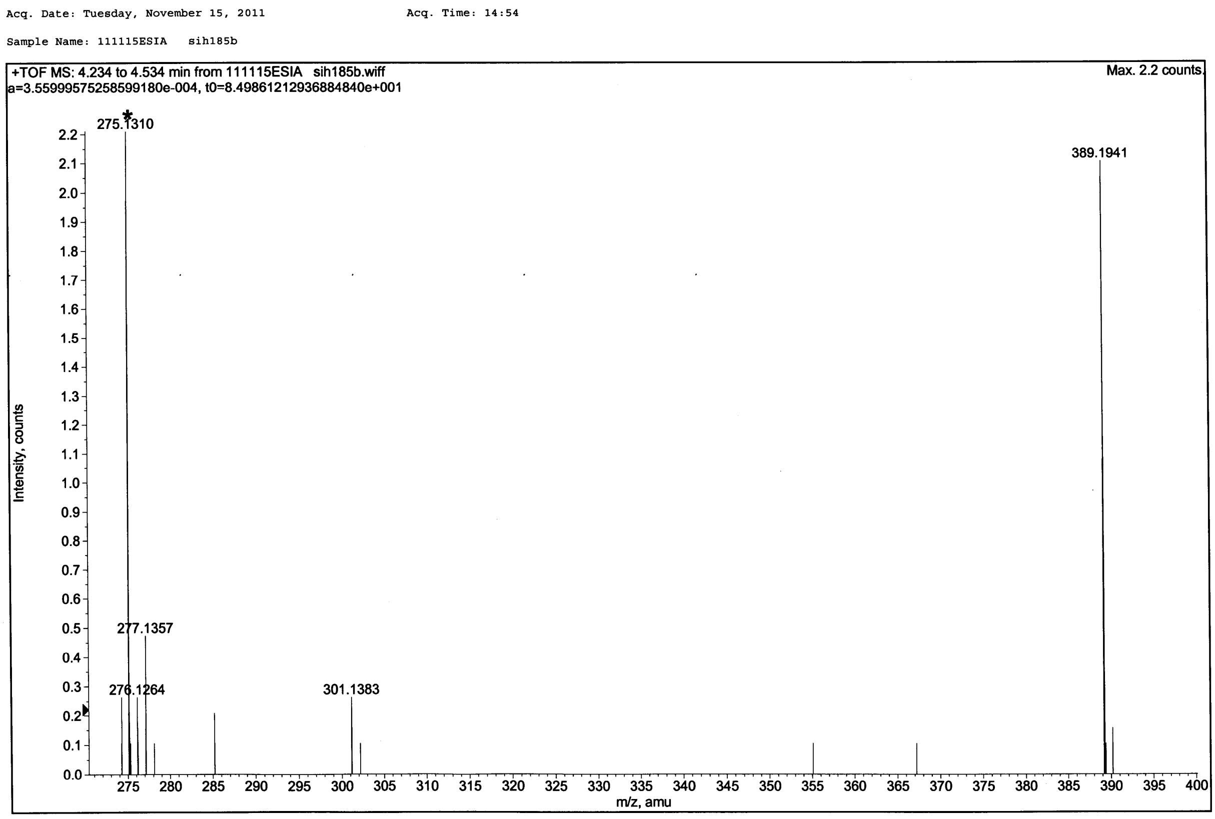

Spectra 9 HRESIMS data of compound **3**

For compound 4

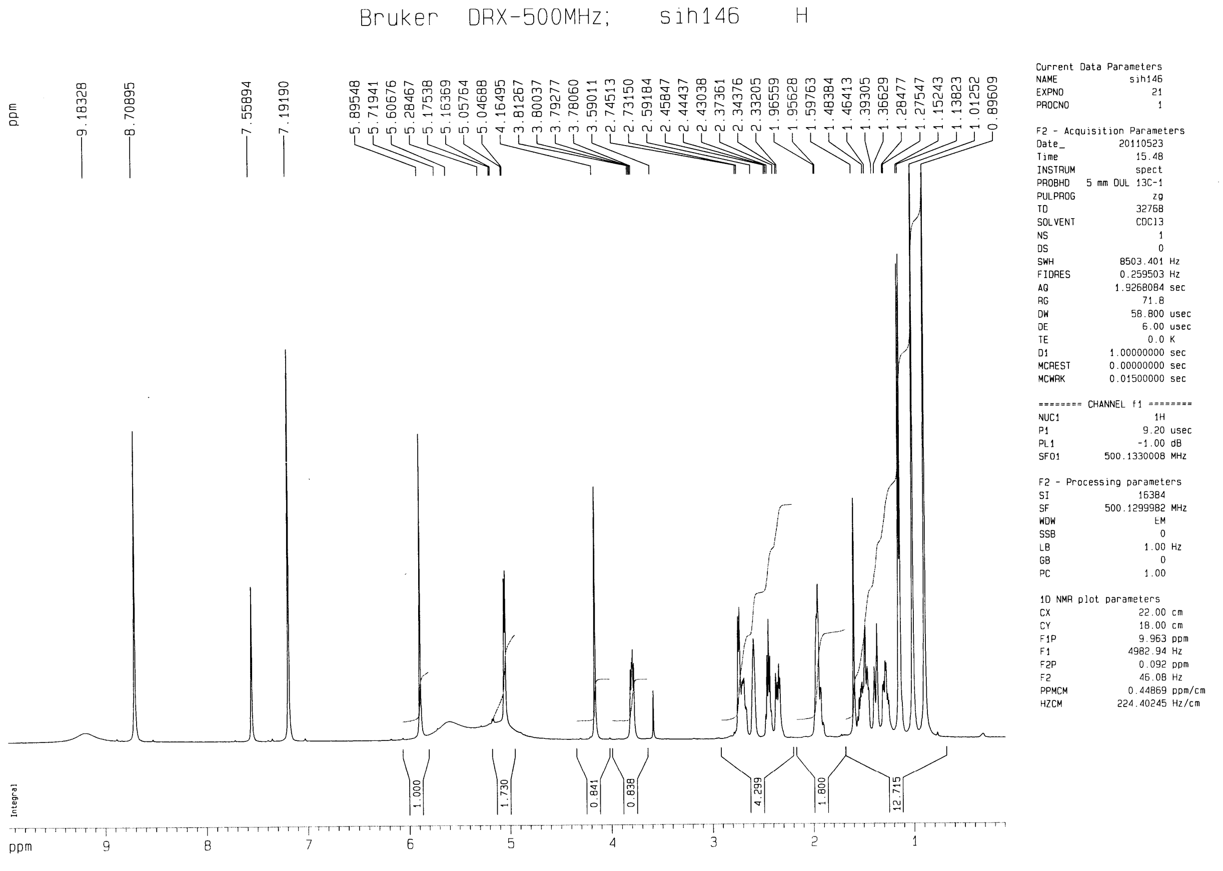

Spectra 10 ^{1}H NMR spectrum of compound **4**

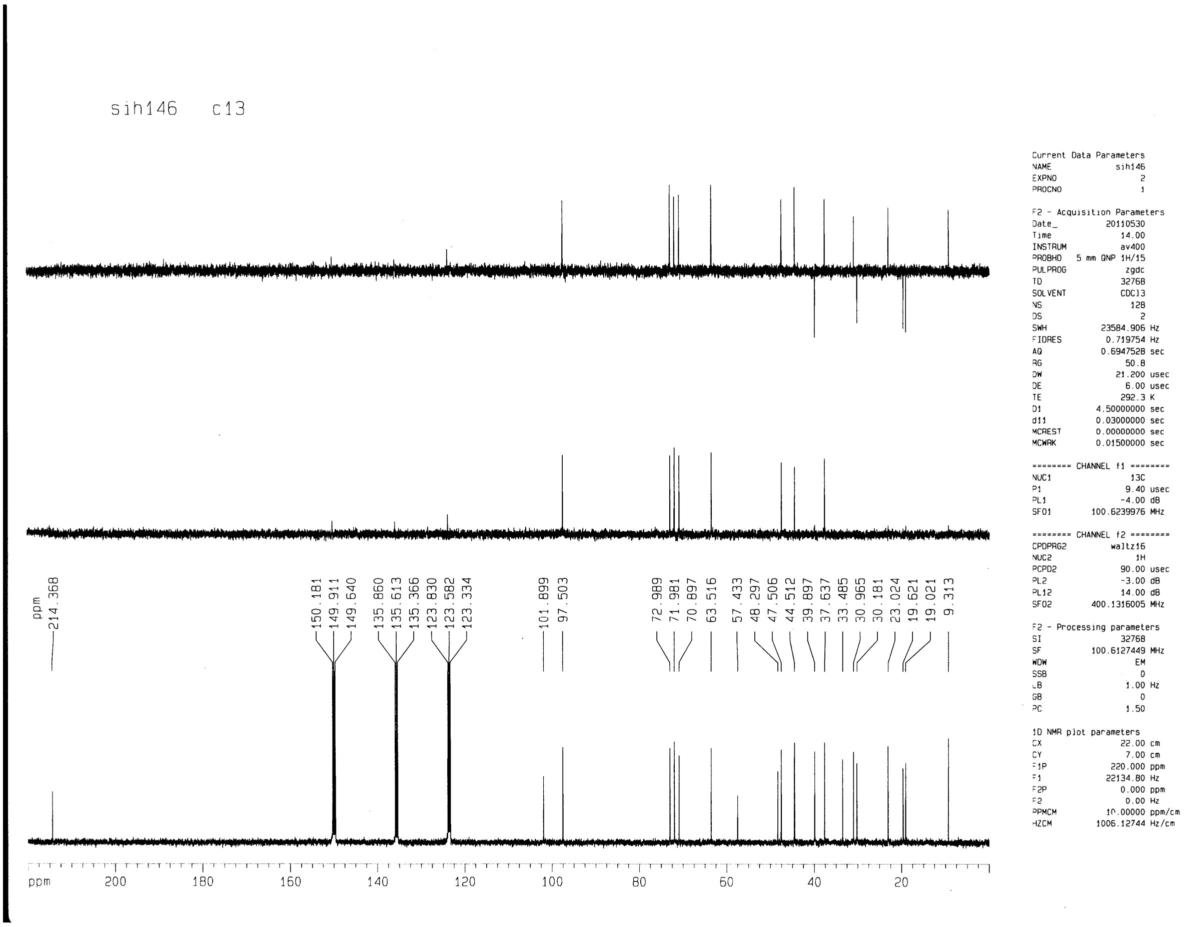

Spectra 11 ^{13}C NMR and DEPT spectra of compound **4**

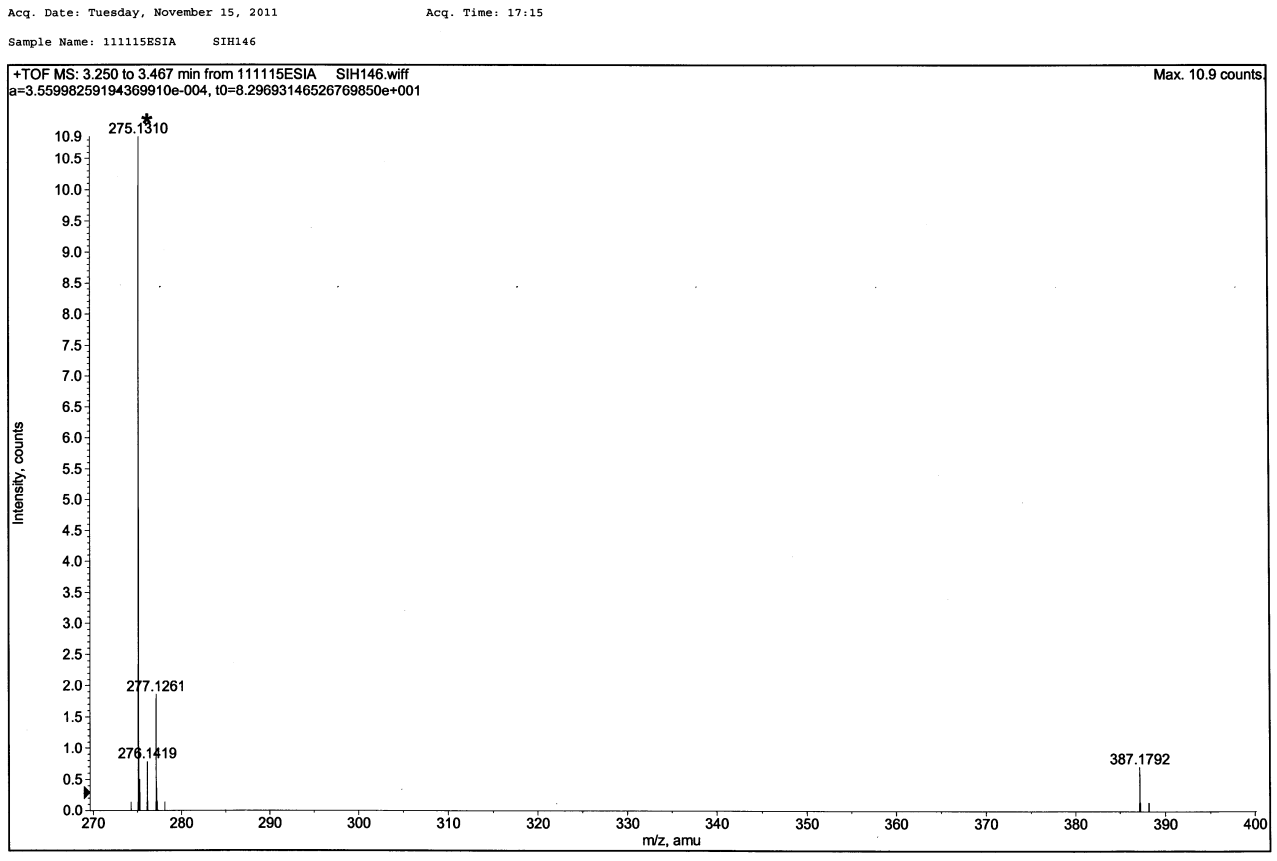

Spectra 12 HRESIMS data of compound **4**
